# A Case Report of Recurrent Acute Laryngeal Dystonia With Different Novel Antipsychotics: Aripiprazole and Olanzapine

**DOI:** 10.7759/cureus.52001

**Published:** 2024-01-10

**Authors:** Anas Ibn Auf, Mohammed A Mohammed, Hasan Wahhas

**Affiliations:** 1 Psychiatry, Erada and Mental Health Complex, Taif, SAU; 2 Psychiatry, Ministry of Health, Jeddah, SAU

**Keywords:** stridor, side effects, mental health, olanzapine, aripiprazole, antipsychotic, laryngeal dystonia

## Abstract

Acute laryngeal dystonia (ALD) is a rare side effect of antipsychotic medications, but it is a life-threatening condition. We are introducing the case of a 49-year-old Saudi single male, who has been known to have schizophrenia for the last 20 years. He developed three attacks of acute laryngeal dystonia owing to different antipsychotic medications. The first was because of haloperidol on a dose of 20 mg a day. After being treated for dystonia and stabilized physically, the patient received oral aripiprazole at a dose of 10 mg a day. Unfortunately, he developed acute laryngeal dystonia, and treatment had to be discontinued. The third attack of dystonia was two months later because of the use of olanzapine in a dose of only 5 mg/day. The patient was finally stabilized on quetiapine with no more side effects. This case highlights the importance of careful monitoring of patients who are receiving antipsychotic medications, even newer ones, to avoid, or treat, such a rare but serious side effect early.

## Introduction

Acute laryngeal dystonia (ALD) is a life-threatening drug-induced dystonic reaction related to the administration of an antipsychotic medication. It is a type of acute dystonic reaction that occurs among other extrapyramidal symptoms characterized by laryngospasm, leading to respiratory compromise that requires immediate medical attention [1].

The first reported cases of ALD were described by Flaherty et al. [2] in 1978 when two individuals developed difficulty breathing shortly after initiating haloperidol. Since then, Christodoulou et al. [3] detailed 28 cases of ALD related to first-generation antipsychotics (FGAs). There are very few cases reported to be associated with second-generation antipsychotics (SGAs) [4-11]. Despite being uncommon, it can be life-threatening with as many as four deaths because of ALD having been reported following the administration of haloperidol [3,11]. We are presenting a patient who developed three different attacks of ALD, which is a rare condition, two of them were because of atypical antipsychotic medications, which is much less. This may be the only reported case with this presentation in the region.

## Case presentation

Mr. A is a 49-year-old Saudi single male, who is a university graduate and was working as a teacher, but he left his work 10 years ago because of his mental illness. He has been known to have schizophrenia for the last 20 years, with a history of multiple hospital admissions and long hospital stays. He received different types of antipsychotic medications throughout his illness, including risperidone, haloperidol, zuclopenthixol, and trifluoperazine, but the patient has poor compliance on medication and medical advice to come for follow-up, and this was one of the reasons of his multiple relapses. There were no documented dystonia or other extrapyramidal side effects in the medical records of this patient.

He was brought to the hospital this time because he showed aggressive behavior; fighting with neighbors, breaking furniture, and setting his house on fire. His condition required hospital admission, and his main features on admission were being agitated, having auditory hallucinations, delusions of persecution, and grandiosity. He was not known hypertensive, diabetic, or any other chronic illness. He was not known to be allergic to any medication or to use illicit psychoactive substances. His physical examination on admission revealed no abnormalities, and also his laboratory investigations include: complete blood count, lipid profile, renal, liver, and thyroid function tests.

The patient received haloperidol 5 mg intramuscular injection once, with lorazepam 2 mg as intramuscular injection to control him in the emergency room. Later in the ward, he continued on oral haloperidol alone, and it was increased gradually to 20 mg/day (in divided doses). After four days on this dose, the patient was noted to suffer while breathing, with harshness of voice and loud voice of inspiration; stridor. He said that "he is unable to breathe normally, he is feeling something occluding his throat." His oxygen saturation (SPO2) decreased to 84%. His pulse was 105 b/min. On auscultation of his chest, air entry was normal to both lungs. Supplemental oxygen therapy was provided, and the patient was referred to the ENT department, where he stayed for one week under assessment, treatment, and observation. The ENT specialist reported no abnormality of the pharynx or respiratory system, suggesting that the condition could be "antipsychotic-induced laryngeal dystonia." The patient was treated with anticholinergic medication; a single dose of benztropine 2 mg intramuscularly then oral doses of 2 mg twice a day for one week. Haloperidol was discontiued.

The treating team in psychiatry did not expect this diagnosis as the patient has a long history of antipsychotics in high doses including haloperidol itself. Despite that, haloperidol was discontinued to avoid the possibility of developing such a serious condition. After a few days, aripiprazole was started at a dose of 10 mg once daily, with regular observation and monitoring for vital signs. One month later, the patient developed the same condition again. He showed shortness of breath with stridor. His blood pressure was 119/70, pulse rate was 110/ min, and his SPO2 was 68%. He has been urgently admitted to the intensive care unit (ICU), intubated, and mechanically ventilated. He spent time there for about two weeks. Again, there was no organic abnormality in the respiratory system, and the diagnosis was antipsychotic-induced laryngeal dystonia.

After the patient became physically well, he began showing active psychotic features, so he was shifted to olanzapine 5 mg once at night with close monitoring and observation. The drug remained at this low dose for the following six weeks despite the presence of psychotic features. Surprisingly, the patient developed a third attack of stridor just before deciding further increase the dose. He was admitted again to the ICU with SPO2: 84%, received the required treatment, and improved.

Later, in the psychiatric ward, the patient was put on quetiapine, which was increased gradually to a dose of 600 mg per day, but he did not develop stridor, laryngeal dystonia, or any other side effects. He achieved partial improvement regarding his psychiatric condition (i.e., he became calm in the ward, his delusions became shakable, and he denied hearing voices anymore). However, it was difficult to discharge him because his social circumstances did not allow for full immediate discharge. The report was prepared after six months of starting quetiapine treatment, with no more attacks of laryngeal dystonia.

## Discussion

ADR is defined as abnormal sustained muscular contractions, developing in association with the use of antipsychotics. These reactions most often involve the muscles of the face, neck, upper limbs, trunk, and even the larynx and rarely involve lower limbs [12]. While it is a rare condition, it should be considered following the start or increase of antipsychotic medications, especially the first generation [13].

Our patient's symptoms are congruent with a diagnosis of ADR related to neuroleptic use. This is because he presented with dyspnoea, clutching of the throat, and sustained spasms of the laryngeal muscles. These symptoms are often misdiagnosed as respiratory distress caused by obstruction, anaphylactic shock, panic attack, etc. [14]. Initially, we sent the patient for an ENT assessment and the possibility of obstructive etiology has been ruled out. Additionally, neuroleptic malignant syndrome was ruled out because of the absence of fever, and no alternation in mental status has been observed. The patient's labs, physical, and imaging did not conclusively offer any alternative explanation to his presentation. Therefore, a diagnosis of ADR related to antipsychotic medication has been made. The possibility of other psychiatric diagnoses causing this condition was discussed; the panic attack was excluded as the condition did not include the important features of intense fear or anxiety. Conversion disorder is unlikely as the patient has a chronic psychotic illness.

This diagnosis was corroborated by the fact that the symptoms developed shortly after the initiation of haloperidol treatment and concurrently after increasing the dose to 20 mg/day. We know from the literature that first-generation antipsychotics carry a burden of extrapyramidal side effects including dystonia [1-4]; however, our patient has an extensive history of antipsychotic treatment including haloperidol without a prior ADR event. A possible justification for not developing ADR before may be because of the patient’s poor adherence to antipsychotic medications in the past.

Although the exact cause of ADR is undetermined, it is theorized that dystonic reactions develop, particularly with first-generation antipsychotics because of their potent D2 receptor antagonistic activity; thus, leading to repetitive stimulation of D1 receptors along with an increase in cholinergic output [4]. Nevertheless, these reactions are unpredictable and are often related to the type of drug, doses taken, and mode of administration, as well as genetic variations and drug interactions. On the other hand, atypical antipsychotics cause fewer extrapyramidal side effects, and they carry a minimal risk for ADR, despite that their use has been implicated in the development of dystonia. In our patient, neither treatment with aripiprazole nor olanzapine has resulted in symptom resolution; in fact, laryngeal dystonia and respiratory distress have not resolved until both were discontinued and the patient was switched to quetiapine.

Moreover, we have found some case reports relating the use of aripiprazole and olanzapine to the development of ADR, but they share similar limitations in concluding the probability of dystonic reaction to be solely caused by their use [4,15,16]. All of the cases reported were compounded by polypharmacy making it harder to attribute symptoms to any particular agent. However, there is a clear temporal association between dose increase - in susceptible individuals - and symptom development. In almost all of these cases, symptoms seized upon discontinuation of the antipsychotic medication, replacing it with one that is less likely to cause ADR like quetiapine, and following the typical management, which largely consists of early recognition, severity assessment, and treatment with either oral, or parenteral anticholinergics, and benzodiazepines. A summary of relevant case reports about aripiprazole-related ADR is presented in Table 1.

**Table 1:** A summary of relevant studies and interventions made. BD: bis in die (twice a day) PO: per os (by mouth) OD: once daily

Author	Goga et al. [4]	Matsuda et al. [15]	Kim et al. [16]
Drug	Aripiprazole 10 mg/day topiramate 25 mg BD	Aripiprazole 18 mg/day	Aripiprazole 20 mg/day
Diagnosis	Bipolar disorder + borderline traits	Paranoid schizophrenia	Schizoaffective disorder
Presentation	Dyspnea dysphonia torticollis	Slurred speech dysphagia	Dystonia of the right neck, torticollis respiratory distress, aspiration pneumonia
Interventions	Benztropine 2 mg bid IM + diazepam 5 mg PO	Biperiden 4 mg/day + discontinuation of aripiprazole	Lorazepam 4 mg/day, benztropine 4 mg/day + discontinuation of aripiprazole
Outcome	No recurrence of laryngeal dystonia upon discontinuation of aripiprazole	Symptoms resolved. Then quetiapine was given up to 400 mg OD with no recurrence	The patient is stabilized on clozapine 200 mg/day, valproic acid 1000 mg/day + diazepam 30 mg/day with no recurrence

In our case, we noticed that dystonic reaction developed relatively late; one month after starting aripiprazole 10 mg and again after six weeks with olanzapine 5 mg. This presentation is atypical since ADR usually develops within hours or a few days. However, our case was not the only one to report this delayed onset; Magnuson et al. reported a similar observation [17]. They documented two cases that took about six weeks to display dystonia symptoms; one of them was a 95-year-old woman who developed dystonia in the form of toricollis, six weeks after starting risperidone [17]. It is unknown whether this delayed onset is a variant from ADR or it can be considered a separate class, but it suggests further investigations to explore the individual, genetic, biological, and environmental factors that may contribute to this atypical presentation.

## Conclusions

ALD, like other rare conditions, is highly likely to be underrecognised. Therefore, a high index of suspicion in psychiatric patients developing acute onset shortness of breath is warranted, particularly in those who have other extrapyramidal side effects. Awareness allows physicians to mitigate the risks early on and prevent morbidity and mortality. Patients who develop ALD need to be promptly diagnosed to give parental anticholinergics and prevent invasive airway management from happening unnecessarily. Awareness of such reactions not only needs to be known in psychiatric settings but also by physicians working in acute care settings and emergency departments. Patients who are starting antipsychotics or increasing their doses must be warned about the possible manifestation of ALD and must receive instructions on how to deal with them if they occur.
